# Management of advanced colorectal cancer: state of the art

**DOI:** 10.1038/sj.bjc.6603233

**Published:** 2006-07-11

**Authors:** M Saunders, T Iveson

**Affiliations:** 1 Christie Hospital NHS Trust, Wilmslow Road, Manchester M20 4BX, UK; 2 Southampton General Hospital, Tremona Road, Southampton SO16 6YD, UK

**Keywords:** colorectal cancer, first-line, 5-fluorouracil, irinotecan, oxaliplatin, targeted agents

## Abstract

Colorectal cancer (CRC) caused over 500 000 deaths worldwide in 2002. Recent advances in the treatment of advanced disease include the incorporation of two new cytotoxic agents, irinotecan and oxaliplatin, into first-line regimens. The concept of planned sequential therapy involving three active agents during the course of a patient's treatment is evolving. Coupled with the integrated use of targeted monoclonal antibodies, we can now expect overall survival rates for advanced disease to exceed 20 months. This review considers current treatments and suggests where future progress may occur.

Globally, over a million cases of colorectal cancer (CRC) were reported in 2002 with the incidence approximately balanced between the sexes. In the same year, over 500 000 deaths were attributed to the disease. There is a 25-fold geographical variation in incidence, presumably as a consequence of dietary factors, with the highest rates of occurrence seen in North America, Australia/New Zealand, Western Europe and in men especially, Japan (Parkin *et al*, 2005).

The majority of CRC patients, perhaps 70–80%, present with apparently resectable localised disease. Surgery, followed by adjuvant therapy for high-risk patients, will be the optimum curative treatment approach in such cases. However, ultimately, approximately half of all diagnosed CRC patients will develop disseminated advanced disease, which in most cases will be fatal. Patients with advanced disease who are sufficiently fit usually receive systemic chemotherapy, first-line, but they may receive best supportive care, surgery (or in the case of rectal tumours, radiotherapy) or a combination of these treatments. Their second-line treatment, if any, is dependent on their response to first-line treatment, disease status and performance status. Chemotherapy for advanced disease is an attempt to alleviate and control symptoms, improve quality of life and improve survival. The aim of this review is to outline the efficacy and safety data for the currently available chemotherapy treatment options for patients with advanced CRC.

## CHEMOTHERAPY IN THE TREATMENT OF PATIENTS WITH ADVANCED CRC

For nearly 50 years, 5-fluorouracil (5-FU) has been the mainstay of chemotherapy treatment for CRC and fluoropyrimidine-based chemotherapy remains a key component of the treatment algorithm for advanced disease, in both the first- and second-line settings. Indeed, until 10 years ago, 5-FU was the only chemotherapy option available to patients. Meta-analyses have shown that chemotherapy for advanced CRC can slow progression and prolong disease survival compared with best supportive care (Colorectal Meta-analysis Collaboration, 2000; Simmonds, 2000). 5-FU in combination with the biomodulator folinic acid (FA) increases the response rate (RR) compared with 5-FU alone (21 *vs* 11%) and confers a small but significant (11.7 *vs* 10.5 months) survival advantage (Thirion *et al*, 2004). Likewise, 5-FU administered as a continuous infusion has superior efficacy when compared with 5-FU administered as an intravenous (i.v.) bolus (RR 22 *vs* 14%) (Meta-analysis Group in Cancer, 1998a). Furthermore, infusional 5-FU regimens have also been shown to be associated with fewer World Health Organization grades 3 and 4 toxicities (Meta-analysis Group in Cancer, 1998b). Generally, physicians in Europe have preferred infusional delivery; with bolus regimens preferred by physicians in the US. However, increasingly there is a shift towards the use of infusional regimens in the US as it is recognised that they are associated not only with a more manageable toxicity profile but also with increased efficacy. The dosing schedules for commonly used bolus and infusional 5-FU-based regimens are summarised in Table 1.

The future of 5-FU-based therapy for the treatment of advanced CRC lies in its combination with newer agents with nonoverlapping toxicity profiles, such as the topoisomerase I inhibitor irinotecan (CPT11, Campto®) and the third-generation platinum compound oxaliplatin (Eloxatin®). Indeed, the infusional 5-FU component of current regimens may be replaced in the future by the new oral fluoropyrimidines, capecitabine (Xeloda®) and uracil-tegafur (UFT®), prodrugs that are designed to mimic infusional 5-FU treatment (Rich *et al*, 2004).

Two key studies demonstrated the superior activity of irinotecan/5-FU/FA compared to 5-FU/FA alone (Douillard *et al*, 2000; Saltz *et al*, 2000), leading to the approval of irinotecan for the first-line treatment of advanced CRC in both the US and Europe in 2000. Likewise, oxaliplatin in combination with infusional 5-FU/FA was approved for use in the first-line setting in Europe in October 1999 and in the US in combination with 5-FU/FA in January 2004.

## IRINOTECAN IN THE FIRST-LINE TREATMENT OF ADVANCED CRC

Three published phase III trials evaluated the role of irinotecan in combination with 5-FU/FA *vs* 5-FU/FA alone in the first-line setting. Two European trials investigated infusional 5-FU/FA regimens (de Gramont and Arbeitsgemeinschaft Internische Onkologie (AIO)); (Douillard *et al*, 2000; Köhne *et al*, 2005), whereas a US trial investigated a bolus 5-FU/FA regimen (Saltz *et al*, 2000). Irinotecan in combination with the *bolus* 5-FU/FA regimen was subsequently shown to be associated with high 60-day mortality levels (Sargent *et al*, 2001) and is now generally the less favoured way of administering this combination.

The addition of irinotecan to 5-FU/FA, irrespective of regimen, conferred a significant clinical benefit, in terms of RR (35 *vs* 22, 39 *vs* 21, 54 *vs* 32%), progression-free survival (PFS: 6.7 *vs* 4.4, 7.0 *vs* 4.3, 8.5 *vs* 6.4 months) and overall survival (17.4 *vs* 14.1, 14.8 *vs* 12.6, 20.1 *vs* 16.9 months) compared with the corresponding 5-FU/FA regimen alone (respectively for Douillard *et al*, 2000; Saltz *et al*, 2000; Köhne *et al*, 2005). Also, although the more recent phase III study of Köhne *et al*, failed to demonstrate a statistically significant improvement in overall survival, the trend of >3 months increase led to one of the longest median overall survival times, to date, in this clinical setting. One can envisage that except for the crossover to irinotecan-based therapy, second-line, in the control 5-FU/FA arms of all three trials, these differences might have been even greater.

The addition of irinotecan to 5-FU/FA did not result in unacceptable toxicity, although it was associated with a higher incidence of grade 3 diarrhoea compared with bolus or infusional 5-FU/FA alone. Importantly, some key 5-FU-associated toxicities: neutropenia, neutropenic fever or sepsis and mucositis were reduced owing to the lower 5-FU dose administered in the combination regimens. The time to deterioration in performance status was also significantly longer for those patients receiving irinotecan/5-FU/FA (Saltz *et al*, 2000).

## OXALIPLATIN IN THE FIRST-LINE TREATMENT OF ADVANCED CRC

Published phase III trials similarly reported that in combination with infusional (de Gramont *et al*, 2000) or chronomodulated (Giacchetti *et al*, 2000) 5-FU/FA, a second new agent, oxaliplatin, was also effective first-line in the treatment of advanced CRC. RRs (51 *vs* 22 and 53 *vs* 16%, respectively) and median PFS times (9.0 *vs* 6.2 and 8.7 *vs* 6.1 months) were improved in the oxaliplatin/5-FU/FA arms compared to the 5-FU/FA alone arms. The improvements in these end points were not, however, accompanied by corresponding significant increases in median overall survival in the oxaliplatin arms, which may be attributable to the use of active salvage therapies for both treatment arms in each study.

Although mild gastrointestinal and haematological side effects are commonly associated with oxaliplatin therapy, the principle dose-limiting toxicities are neurotoxicity (which may be acute or chronic) and neutropenia (Grothey and Goldberg, 2004). Acute neurotoxicity (paresthesias or dysesthesias), although frequently seen, is generally transient and mild (Gamelin *et al*, 2002). However, after several cycles of oxaliplatin therapy, a late-onset cumulative sensory neuropathy may occur. This side effect typically improves rapidly with the discontinuation of oxaliplatin treatment (Grothey, 2003).

## RELATIVE EFFICACY OF FIRST-LINE COMBINATIONS

Comparison of oxaliplatin combined with an infusional 5-FU/FA regimen (FOLFOX) with irinotecan in combination with bolus 5-FU/FA (IFL) in the US intergroup study (Goldberg *et al*, 2004) suggested that the FOLFOX combination was superior in terms of first-line efficacy. These results formed the basis of the Food and Drug Administration approval of FOLFOX as a first-line therapy for advanced CRC in January 2004. However, the bolus 5-FU component of the IFL regimen is known to be inferior in terms of efficacy to the infusional 5-FU component of FOLFOX (Meta-analysis Group in Cancer, 1998a) and so conclusions cannot be safely drawn from this study concerning relative efficacy of FOLFOX and irinotecan in combination with infusional 5-FU/FA (FOLFIRI).

However, the relative efficacy of these two regimens was directly compared in the randomised Tournigand trial (Tournigand *et al*, 2004). Although this study was primarily designed to investigate whether the sequence of administration of FOLFOX and FOLFIRI was important in terms of second PFS (the time from randomisation until disease progression after second-line therapy), it provides an important insight into the relative efficacies of these combinations in both the first- and second-line settings. Patients were randomised to one of the two treatment arms. Those in arm A received FOLFIRI (irinotecan 180 mg m^−2^ and FA 200 mg m^−2^ on day 1, followed by bolus 5-FU 400 mg m^−2^ and continuous 5-FU, 2400–3000 mg m^−2^, by 46-h infusion) until disease progression or unacceptable toxicity, at which time they crossed over to receive oxaliplatin (100 mg m^−2^ on day 1) in combination with the same modified de Gramont (MdG) 5-FU/FA regimen (FOLFOX6). Conversely, the patients assigned to arm B received FOLFOX6 until disease progression, at which time they crossed over to receive FOLFIRI.

The median overall survival was 21.5 months for the 109 patients in arm A (FOLFIRI followed by FOLFOX6) and 20.6 months for the 111 patients assigned to arm B (FOLFOX6 followed by FOLFIRI), leading to the conclusion that the two regimens were essentially indistinguishable in terms of efficacy. However, it was noted that a significantly higher number of patients in arm B had their metastatic disease rendered resectable (*P*=0.02). As expected, the toxicity profiles in first-line therapy of the two regimens were different, with grade 3/4 mucositis and nausea/vomiting more common with FOLFIRI and grade 3/4 neutropenia and neurosensory toxicity more frequent with FOLFOX6. In particular, the number of patients who had to stop oxaliplatin therapy before tumour resistance owing to neurotoxicity was a limitation of the study. The most important observation from the Tournigand study was therefore that median survival was in excess of 20 months for both arms when the two combinations were used sequentially (effectively, the use of three active drugs during the course of the patient's treatment).

**Figure 1:**
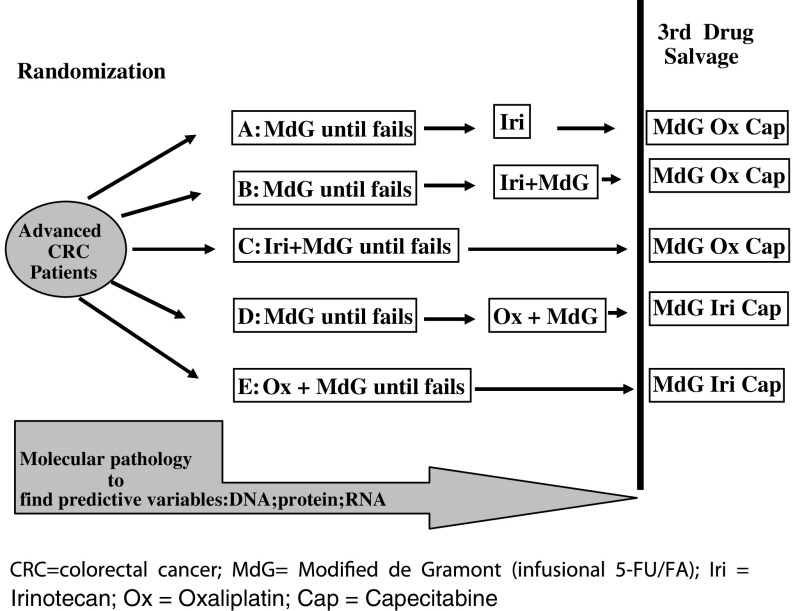
Randomisation for the MRC CR08 FOCUS trial.

The UK MRC CR08 FOCUS trial was designed to assess the role of irinotecan or oxaliplatin combined with the modified MdG infusional 5-FU/FA regimen, in the first- and second-line treatment of patients with advanced CRC. Patients (2135) with good performance status were randomly allocated to one of five treatment arms (Figure 1): staged single-agent therapy (arm A), staged combination therapy (arms B and D) or first-line combination therapy (arms C and E). Only slight (nonsignificant) increases in overall survival were seen with each combination therapy (hazard ratios: 0.86–0.96) over the staged single-agent arm (Seymour, 2005). This suggests that staged combination therapy may provide an alternative treatment strategy for those patients unable to tolerate first-line combinations.

Interestingly, rather than use of irinotecan- and oxaliplatin-based regimens sequentially, impressive efficacy data have been reported from a randomised phase III study in which biweekly irinotecan, oxaliplatin and infusional 5-FU/FA (FOLFOXIRI) was compared first-line to FOLFIRI in 244 previously untreated advanced CRC patients (Falcone *et al*, 2006). FOLFOXIRI was assessed as feasible with manageable toxicities. After a median follow-up of 14 months, RRs were significantly higher (66 *vs* 41%, *P*=0.0002) and PFS was significantly longer (9.8 *vs* 6.8 months, *P*=0.0002) in the FOLFOXIRI arm. Full efficacy data are awaited with interest.

In summary, considering all of the published data and acknowledging that the side-effect profiles for each drug are different, it would appear that at least for the moment, a clear case cannot be made for the preferential use of either irinotecan or oxaliplatin in first-line combinations (Punt, 2005). However, other factors such as the relative efficacy of these drugs in the adjuvant setting might in the future direct which agent is used first-line.

## THE INFLUENCE OF SECOND-LINE THERAPY ON SURVIVAL

The importance of effective second-line therapy was first highlighted by the early phase III trials in which single-agent irinotecan was used as salvage therapy (Cunningham *et al*, 1998; Rougier *et al*, 1998). The FOLFIRI/FOLFOX crossover in the Tournigand study achieved the longest survival recorded to date, for a phase III trial in advanced CRC. An interesting question is how much did crossover therapy contribute to the clinically significant benefits in survival of over 2–3 months reported for the phase III trials of irinotecan/5-FU/FA in the first-line setting (Douillard *et al*, 2000; Saltz *et al*, 2000; Köhne *et al*, 2005)?

It should be noted that only 50–60% of patients may actually be well enough to receive such second-line therapy, and consequently, for a significant number of patients, the choice of an optimum first-line regimen is critical. However, if we compare the survival data for trials of irinotecan/5-FU/FA and oxaliplatin 5-FU/FA combinations (Table 2), we can see that overall survival appears to increase in proportion to the number of patients who are able to receive second-line therapy.

Significantly, Grothey and Sargent (2005), in a recent analysis of 21 arms of 11 phase III trials, have shown overall survival to be significantly correlated with the percentage of patients who received all three active drugs (irinotecan, oxaliplatin, 5-FU/FA) during the course of their disease (*P*=0.0001), and not simply with the percentage of patients who received any second-line chemotherapy (Grothey *et al*, 2004). Although the first-line use of a chemotherapy doublet over 5-FU/FA alone was not associated with a significant efficacy benefit in the expanded analysis (Grothey and Sargent, 2005), the early use of combination therapy increases the likelihood that a patient will receive all three active drugs during their treatment.

## HEPATIC RESECTION

Approximately half of CRC patients develop liver metastases during the course of their disease. Potentially curative surgical resection of secondary tumours is an option for less than 20% of such patients. In the remainder, the hepatic lesion is either ill sited, too large or multinodular, and therefore deemed to be unresectable. In a proof-of-principle retrospective study, evaluating a chronomodulated schedule of 5-FU/FA and oxaliplatin, Giacchetti *et al* (1999) demonstrated that first-line cytotoxic chemotherapy had downsized a significant fraction of unresectable disease to operability. Specifically, following the treatment of 151 patients with liver-only, previously inoperable metastatic disease, 77 were subsequently resected with curative intent. Median overall survival in this group was 48 months against 15.5 months in the nonoperated patients.

A recent analysis of all published trials and retrospective studies that had reported tumour response and resection rates of initially unresectable hepatic lesions highlighted how effective this approach can be in advanced CRC. For patients with inoperable metastases confined to the liver (selected patients), resection rates after first-line chemotherapy with a range of regimens were 24–54%, and for all patients with advanced disease (unselected patients), the rate was 1–26%. A highly significant correlation between tumour response and resection rates was seen for both selected and unselected patients, indicating that regimens that produce the highest RRs are likely to be those that allow the highest hepatic resection rates (Folprecht *et al*, 2006). One factor that should be considered in such evaluations is that a partial response to treatment is often sufficient and preferable for the facilitation of potentially curative liver resection.

The recent phase II study of Alberts *et al* (2005) confirmed the efficacy of FOLFOX4 in the neoadjuvant setting, with 25 of the 42 (60%) assessable patients with nonoptimally resectable liver-only metastatic disease showing a reduction of tumour burden and 17 (40%) patients subsequently undergoing surgery following a median of 6 months of chemotherapy. A high recurrence rate of 71% after a median follow-up of 22 months was observed in this study for patients who underwent complete resection. It is to be hoped that the addition of targeted agents to FOLFOX might further improve efficacy in this setting. Consideration is also being given to the question of whether perioperative FOLFOX4 is feasible and clinically effective in the randomised phase III EORTC 40983 study. Early results suggest that such treatment can be administered safely and without interfering with the timing of surgery (Nordlinger *et al*, 2005). Efficacy data are awaited with interest.

## CHOICE OF FIRST-LINE TREATMENT FOLLOWING ADJUVANT THERAPY

There is, however, a change in the dynamics of the treatment for CRC emerging. Oxaliplatin/5-FU/FA has been shown to prolong disease-free survival in patients undergoing curative resection for stage II and III CRC compared with 5-FU/FA alone (Andre *et al*, 2004), and has consequently been approved for the adjuvant therapy of patients with stage III CRC. It is likely that this will in turn lead to the increased use of irinotecan/5-FU/FA first-line for advanced disease. This possibility is further strengthened by the disappointing data from adjuvant therapy trials of irinotecan/5-FU/FA combinations. In particular, after a median follow-up of 32 months, the PETACC3 study has so far failed to show a statistically significant survival benefit from adding irinotecan to an infusional 5-FU/FA regimen in stage III colon cancer patients (Van Cutsem *et al*, 2005). As oxaliplatin-based combination adjuvant therapy may be more appropriate for patients with curatively resected colon cancer, the likelihood of irinotecan being used in the first-line setting upon relapse is increased. However, if the disease-free interval is prolonged after oxaliplatin-based adjuvant therapy, then rechallenging a patient with oxaliplatin when they re-present with metastatic disease could be considered. However, if a patient is troubled by persistent neuropathy after adjuvant oxaliplatin, then it would not be appropriate to reintroduce this agent and therefore irinotecan-based therapy may be preferred.

## ORAL FLUOROPYRIMIDINE THERAPY

It is possible that in the future, the orally active prodrug, capecitabine, will increasingly replace 5-FU in combined regimens. Capecitabine is preferentially activated in neoplastic tissue by a process that exploits the high thymidine phosphorylase enzyme activity in tumours, thereby providing continuous targeted fluoropyrimidine exposure without the inconvenience for the patient of infusional delivery (Rich *et al*, 2004). Phase III trials in advanced CRC have shown an improved safety profile over bolus 5-FU/FA (Mayo Clinic regimen) (Van Cutsem *et al*, 2004a) and a recent randomised crossover trial has confirmed a high level of patient preference for oral over i.v. fluoropyrimidine therapy (Twelves *et al*, 2006). Capecitabine was also shown in the adjuvant setting to be at least equivalent in terms of efficacy to bolus 5-FU/FA, with a significant increase in relapse-free survival in the capecitabine *vs* 5-FU/FA group (*P*=0.04) (Twelves *et al*, 2005). However, one recent report of a randomised phase III trial of first-line infusional 5-FU/FA/oxaliplatin (FUFOX) *vs* capecitabine plus oxaliplatin (CAPOX) has so far failed to exclude the inferiority of the CAPOX regimen (Kubicka *et al*, 2006). Indeed, it has been argued that combinations of capecitabine with oxaliplatin or irinotecan are currently probably best placed within clinical trial settings (Köhne and Folprecht, 2006).

## TARGETED AGENTS

As our knowledge of tumour biology and genetics matures, a range of agents that interact with novel disease-associated targets are emerging into the clinical setting. Two drugs already approved for the treatment of CRC are the monoclonal antibodies: cetuximab (Erbitux®), which binds to and inhibits activation of the epidermal growth factor receptor (EGFR) (Li *et al*, 2005), and bevacizumab (Avastin®), which binds vascular endothelial growth factor (VEGF-A), thereby interfering with signalling through the VEGF-1 and -2 receptors and inhibiting angiogenesis (Hicklin and Ellis, 2005). Most of the mature data relating to the efficacy of targeted agents in CRC treatment have been derived using irinotecan combinations. However, considerable evidence is now emerging that, as expected, these targeted drugs are not agent specific and that they are likely to improve the efficacy of both irinotecan and oxaliplatin combinations.

### Cetuximab

Cetuximab has been approved in both Europe and the US for use in combination with irinotecan as second-line therapy in CRC patients who have failed prior irinotecan treatment. In the pivotal study, Cunningham *et al* (2004) randomised 329 previously treated patients who had progressed during or immediately following irinotecan-based therapy into two groups, which received either cetuximab plus irinotecan (218) or cetuximab (111). They demonstrated a higher RR (22.9 *vs* 10.8%) and an increase in the median time to progression (4.1 *vs* 1.5 months) for the combination therapy group compared to the monotherapy group.

Cetuximab was subsequently investigated first-line in combination with irinotecan/infusional 5-FU/FA (Rougier *et al*, 2004; Folprecht *et al*, 2006). Initial results showed these combinations to be safe with promising activity. Early data also suggested that cetuximab combined with FOLFOX is an active and safe combination in the first-line treatment of CRC (Polikoff *et al*, 2005). Indeed, the confirmed RR of 72% to cetuximab/FOLFOX4 in one first-line phase II trial is one of the highest so far recorded for this setting and resulted in a resection rate for initially inoperable metastatic disease of 23% in the unselected patient series (Diaz-Rubio *et al*, 2005).

Cetuximab does not appear to increase the intensity or frequency of the characteristic side effects of cytotoxic chemotherapy. The most common cetuximab-related adverse event reported is the development of an acne-like rash. This class effect of EGFR inhibitors is generally manageable (Segaert *et al*, 2005) and may be indicative of a response to cetuximab (Van Cutsem *et al*, 2004b).

### Bevacizumab

Bevacizumab in combination with irinotecan/bolus 5-FU/FA has been approved for the first-line therapy of patients with advanced CRC based on the data from the Hurwitz trial (Hurwitz *et al*, 2004). Patients in this study (813) were randomised into two groups: irinotecan/5-FU/FA/bevacizumab (402) *vs* irinotecan/5-FU/FA plus placebo (411). There was an improved median duration of survival (20.3 *vs* 15.6 months), an increased RR (44.8 *vs* 34.8%) and a longer median progression-free survival (10.6 *vs* 6.2 months) in the bevacizumab over the placebo arm.

The second-line use of bevacizumab in combination with oxaliplatin was subsequently explored in the randomised phase III ECOG 3200 study, which investigated the efficacy and safety of bevacizumab alone and in combination with FOLFOX4 *vs* FOLFOX4 alone in 829 CRC patients previously treated with irinotecan and a fluoropyrimidine (Giantonio *et al*, 2005). After a median follow-up period of 18.7 months, there was a statistically significant advantage in the bevacizumab/FOLFOX4 arm *vs* the FOLFOX arm for both overall survival and PFS (12.5 *vs* 10.7 and 7.4 *vs* 5.5 months).

The first-line use of bevacizumab has also been investigated in the sequential randomised TREE-1 and TREE-2 trials. The TREE-1 study was initially designed to evaluate the efficacy and safety of three different oxaliplatin and fluoropyrimidine combinations: FOLFOX, oxaliplatin/bolus 5-FU/FA (bFOL) and capecitabine and oxaliplatin (CapOx). Following the regulatory approval of bevacizumab in 2004, TREE-2 was initiated with the aim of evaluating the benefits of bevacizumab in combination with the same regimens. Cross-study comparison suggested that the addition of bevacizumab improved the RR and median time to progression in each arm of the study (Hochster *et al*, 2005, 2006). Overall survival data are eagerly awaited.

The use of bevacizumab has been associated with a low level of gastrointestinal perforation events (Kozloff *et al*, 2006) and some concern has been expressed as to whether anti-VEGF therapy might inhibit wound healing (Scappaticci *et al*, 2005). This concern led to the recommendation that a patient should not undergo elective hepatic resection during or within 8 weeks of bevacizumab treatment (Ellis *et al*, 2005).

## FUTURE DIRECTIONS: ‘PERSONALISED’ THERAPY

New targeted agents directed against specific proteins and pathways are likely to continue to improve our ability to treated advanced CRC. In addition, considerable evidence indicating that the expression levels or functionality (coding sequence variation) of certain genes can affect either how a tumour or a patient responds to a particular drug is also accumulating. Typing such variation before treatment begins may allow the physician in the future to select on a case-by-case basis the most appropriate treatment agents or dose levels (reviewed by Russo *et al*, 2005). An example of the potential of this approach is provided by recent data suggesting that constitutional allelic variation in UDP-glucuronosyltransferase, a protein involved in the metabolism of irinotecan, may predict response and toxicity in patients with advanced CRC treated with capecitabine/irinotecan (Carlini *et al*, 2005). Prospective studies are clearly required to validate such observations and to extend their scope to include wider considerations of other ADME (absorption, distribution, metabolism, excretion) genes, for example, the ATP-binding cassette transporters, which can contribute to irinotecan efflux (Rhodes *et al*, 2005).

The use of such approaches is clearly some way in the future. However, the strong possibility that the performance of existing effective treatments might be further improved by the consideration of the genetic background of the patient and/or the genetic/epigenetic status of the tumour is an enticing prospect. If we are to realise this potential, the collection and analysis of appropriate biological material from trial participants needs to be routinely carried out in current and future studies. Subsequent analyses will be facilitated by the progressively decreasing costs of genomics and genotyping technologies.

## CONCLUSIONS

The use of irinotecan or oxaliplatin in combination with 5-FU/FA first-line is consistently associated with a clinically significant improvement in survival. If oxaliplatin is used more widely in the adjuvant setting, as seems likely given the data from recent trials, the use of irinotecan as first-line therapy is likely to increase in the future. The use of three cytotoxic drugs (irinotecan, oxaliplatin and 5-FU/FA) during the course of a patient's treatment has been shown to maximise survival.

**Figure 2:**
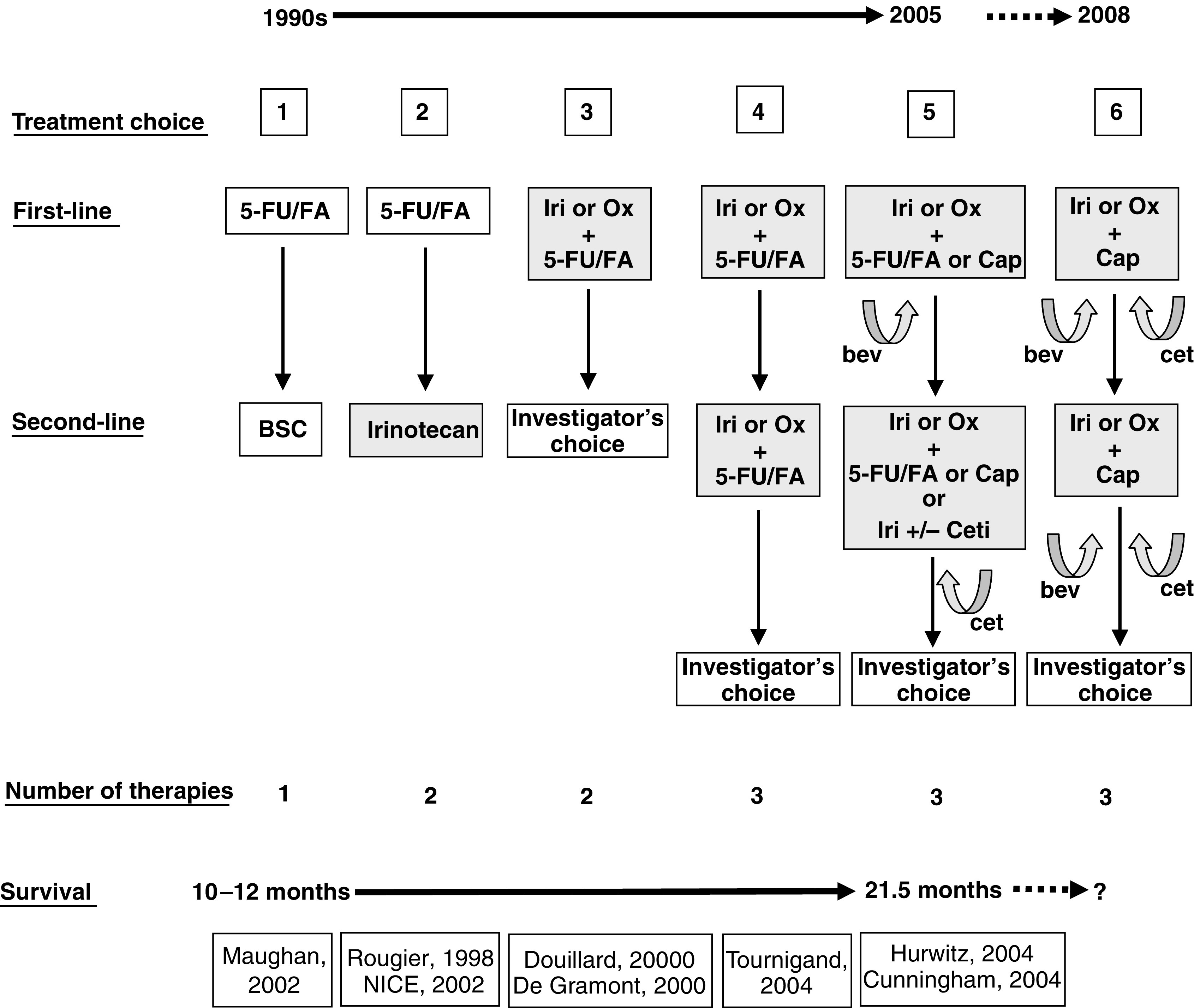
Expansion of the treatment choices for patients with advanced CRC (Rougier *et al*, 1998; de Gramont *et al*, 2000; Douillard *et al*, 2000; Maughan *et al*, 2002; Cunningham *et al*, 2004; Hurwitz *et al*, 2004; Tournigand *et al*, 2004) Iri=irinotecan; Ox=oxaliplatin; Cap=capecitabine; bev=bevacizumab; cet=cetuximab; BSC=best supportive care.

The selection of the optimum treatment regimen for CRC remains difficult, with a number of effective choices available to patients and clinicians (Figure 2). The number of such alternatives is likely to increase over the coming years. Currently, the optimum first-line approach is to use either FOLFOX or FOLFIRI combined with a biological agent such as bevacizumab. The alternative regimen can thereafter be used as second-line treatment, again combined with a different biological agent, such as cetuximab. Although maybe not yet seen as an optimal marketing strategy by the pharmaceutical sector, as our knowledge of predictive tumour markers increases and as a more diverse range of targeted drugs becomes available, further dramatic progress in our ability to treat advanced CRC could lie in a more individualised and scientific process of treatment selection for the patient.

## Figures and Tables

**Table 1:** Summary of frequently used bolus and infusional 5-FU/FA regimens

**Regimens**	**Doses and schedules of administration**
Mayo Clinic Regimen (Saltz *et al*, 2000)	5-FU (425 mg m^−2^ day^−1^) i.v. bolus+FA (20 mg m^−2^ day^−1^) daily days 1–5 every 4 weeks
Saltz (modified Roswell Park regimen) (Saltz *et al*, 2000)	5-FU (500 mg m^−2^ day^−1^) i.v. bolus+FA (20 mg m^−2^ day^−1^) weekly for 4 weeks every 6 weeks
De Gramont (de Gramont *et al*, 1997)	FA (200 mg m^−2^) 2 h+5-FU (400 mg m^−2^) i.v. bolus+22 h infusion 5-FU (600 mg m^−2^) for 2 consecutive days every 2 weeks
Modified de Gramont (Leonard *et al*, 2002)	FA (175–200 mg m^−2^) 2 h+5-FU (400 mg m^−2^) i.v. bolus+46 h infusion 5-FU (2400–3000 mg m^−2^) for 2 consecutive days every 2 weeks
AIO (Köhne *et al*, 2005)	5-FU (2600 mg m^−2^) 24 h CI+FA (500 mg m^−2^), weekly × 6, every 7 or 8 weeks

AIO=Arbeitsgemeinschaft Internische Onkologie (German Co-operative Group); CI=continuous infusion; FA=folinic acid; 5-FU=5-fluorouracil; i.v.=intravenous.

**Table 2:** Survival data for patients who received irinotecan/5-FU/FA or oxaliplatin/5-FU/FA combination therapy first-line: influence of second-line therapy on survival

**First-line regimen**	**Patients receiving therapy second-line (%)**	**Patients receiving three active drugs^a^ (%)**	**Median OS (months)**	**Study data**
Irinotecan+bolus 5-FU/FA	52	5	14.8	Saltz *et al* (2000)
Irinotecan+bolus 5-FU/FA	67	24	15.0	Goldberg *et al* (2004)
Irinotecan+inf. 5-FU/FA	39.4	15.7	17.4	Douillard *et al* (2000)
Irinotecan+AIO	56	52	20.1	Köhne *et al* (2005)
Irinotecan+inf. 5-FU/FA	81	74	21.5	Tournigand *et al* (2004)
FOLFOX	58	29.5	16.2	De Gramont *et al* (2000)
FOLFOX	75	60	19.5	Goldberg *et al* (2004)
FucOX^a^	NA	NA	19.4	Giacchetti *et al* (2000)
FOLFOX	74	62	20.6	Tournigand *et al* (2004)
FUFOX (bolus 5-FU/FA (Mayo Clinic))	81	67.5	20.4	Grothey *et al* (2004)

AIO=AIO infusional 5-FU/FA; FA=folinic acid; 5-FU=5-fluorouracil; inf=infusional; OS=overall survival; NA=not applicable.

a FUcOX=chronomodulated 5-FU/FA plus oxaliplatin. ^a^(5-FU/FA, irinotecan and oxaliplatin) during first- and second-line treatment.
